# Patients as consumers of health care in South Africa: the ethical and legal implications

**DOI:** 10.1186/1472-6939-14-15

**Published:** 2013-03-21

**Authors:** Kirsten Rowe, Keymanthri Moodley

**Affiliations:** 1 Centre for Medical Ethics and Law, Department of Medicine, Faculty of Medicine and Health Sciences, Stellenbosch University, Tygerberg Campus, Cape Town, South Africa

**Keywords:** Patients, Consumers, Health care, Consumerism, Commodification, Autonomy, Rights, Ethical, Legal, South African

## Abstract

**Background:**

South Africa currently has a pluralistic health care system with separate public and private sectors. It is, however, moving towards a socialised model with the introduction of National Health Insurance. The South African legislative environment has changed recently with the promulgation of the Consumer Protection Act and proposed amendments to the National Health Act. Patients can now be viewed as consumers from a legal perspective. This has various implications for health care systems, health care providers and the doctor-patient relationship.

**Discussion:**

Calling a recipient of health care a ‘consumer’ as opposed to a ‘patient’ has distinct connotations and may result in differential behaviour. Labels reflect the ideals of the context in which they are used. Various models of the doctor-patient relationship exist and different metaphors have been used to describe it. Increasingly there are third parties involved within the doctor-patient relationship making it more difficult for the doctor to play the fiduciary role. In certain parts of the world, there has been a shift from a traditional paternalistic model to a consumerist model. The ethical implications of the commodification of health care are complex. As health care becomes a ‘product’ supplied by the health care ‘provider’, there is the risk that doctors will replace professional ethics with those of the marketplace. Health care is a universal human need and cannot be considered a mere commodity. In modern medical ethics, great emphasis is placed on the principle of respect for patient autonomy. Patients are now the ultimate decision-makers. The new Consumer Protection Act in South Africa applies to consumers and patients alike. It enforces strict liability for harm caused by goods and services. Everyone in the supply chain, including the doctor, can be held jointly and severally liable. This may lead to enormous challenges in health care delivery.

**Summary:**

Viewing patients as consumers may be detrimental to the doctor-patient relationship. While it facilitates an emphasis on respect for patient autonomy, it inadvertently results in the commodification of health care. The new legislative environment in South Africa promotes the protection of patient rights. It may, however, contribute to increased medical litigation.

## Background

South Africa is home to approximately 50.5 million people from diverse cultural and religious backgrounds [1]. Eleven official languages are recognised in celebration of the country’s rich diversity. South Africa is a land of contrasts and paradoxes. Although the country enjoyed a peaceful transition from the apartheid era to the current constitutional democracy, the vast inequalities that were entrenched due to the previous discriminatory, racially-based legislation are part of the legacy that remains. According to the World Bank, the gap between rich and poor may actually be widening. Despite being classified as an upper middle income country, South Africa has the highest level of inequality in the world with a GINI coefficient of 0.70 - the poorest fifth of the population accounting for 2% of the country’s income and consumption, and the richest fifth for 72% [1,2].

Currently South Africa has a pluralistic, transitional health care system which reflects this inequality. The two-tiered health care system has separate public and private streams [3]. The public sector, funded by general tax, is based on a district health system approach with its emphasis on primary health care. Although there are out-of-pocket payers who can self-fund primary health care in the private sector, but rely on the state for secondary and tertiary care, 68% of the population depend entirely on the public health sector. Only 16% of citizens can afford private medical scheme cover and are able to access private health care exclusively, yet this portion of the population accounts for up to 45% of the total national health expenditure. The private sector also enjoys a much more favourable health care provider to patient ratio. The introduction of National Health Insurance (NHI) may help to achieve greater equity [4].

From a sociological perspective, the choice of labelling patients as health care consumers is highly significant. In light of the labelling or social reaction theory, a person’s sense of identity and behaviour can be influenced by the titles assigned to him or her. However, sometimes the terms reflect the times. The current historical context, including the present political and economic ideologies, has an impact on the type of health care system adopted which in turn has ramifications for participants. A consumerist model may view patients as consumers, whereas a socialised model like the one South Africa is moving towards with the NHI may view patients as beneficiaries or as entitled ‘users’ - the term employed in the National Health Act No 61 of 2003 [5].

Historically, various metaphors have been adopted to describe the doctor-patient relationship ranging from paternalism to consumerism. The role of fiduciary with its emphasis on accountability is particularly important in the age of patient autonomy, empowerment and protection of the rights of the patient and the consumer. The ethical implications of patients being viewed as consumers are potentially problematic. If health care is treated as a commodity, this may result in the replacement of professional ethics with business ethics. In a developed context, in particular, patients may abuse their autonomy. In a developing context where people were previously oppressed, as has occurred in much of South Africa, empowering patients as consumers might be a step in the right direction. It may, however, be difficult for disempowered state patients who are receiving free care to view themselves as “consumers”. Recent legislative changes in South Africa, including the new Consumer Protection Act and the proposed amendments to the National Health Act, will result in patients being viewed as consumers from a legal perspective. These laws should create a favourable regulatory environment for the protection of patient and consumer rights. This paper will discuss the ethical and legal implications of patients being viewed as consumers of health care.

## Discussion

### The connotations and sociological impact of labelling

Do the labels we attach to various roles in society really matter? Is there a difference between calling a recipient of health care a ‘patient’ and calling him or her a ‘health care consumer’? According to the labelling or social reaction theory, the perception of labels leads to differential behaviour [6]. Although changing labels or titles reflect changes that have occurred in society over time, sometimes it is also the new label itself that helps to alter thinking about a particular role in society. Current standards in ethics and law encourage active patient participation and empowerment, and recognition of the patient’s right to autonomy or self-determination with the doctrine of informed consent taking a central place in the modern doctor-patient relationship. A study by The Department of Health Policy, Management and Evaluation at the University of Toronto aimed to clarify preferred labels for those receiving health care in light of the shift away from the old doctor-patient relationship where the patient was passive and the doctor was the main decision-maker. Deber et al. suggest that an associated adjustment of the language used might be appropriate. Potential new labels for a ‘patient’ include ‘partner’, ‘survivor’, ‘consumer’, ‘customer’, ‘client’, ‘purchaser’ and ‘user’ [7]. Each term has its own implications. Collectively, the terms ‘consumer’, ‘customer’, and ‘client’ imply that health care is a commodity or product to be managed in the market. The term ‘user’ is employed in the South African National Health Act No 61 of 2003 and defined in Section 1 as a person who receives treatment in a health establishment or someone using health services [5].

The English term ‘patient’ comes from the Latin present participle ‘patiens’ meaning ‘suffering’. The Oxford Dictionary defines the noun ‘patient’ as ‘a person receiving or registered to receive medical treatment’. The term can also be used as an adjective to describe the quality of having or showing patience, in other words ‘able to accept or tolerate delays, problems, or suffering without becoming annoyed or anxious’ [8]. In contrast, the term ‘consumer’ comes from the Latin ‘consumere’, the infinitive meaning ‘to take up completely’ [7]. It is defined as ‘a person who purchases goods and services for personal use’ or ‘a person or thing that eats or uses something’ [8]. The two terms have distinct connotations.

### The role of the historical, political and economic context and the health care system

Terms reflect the times and the predominant ideology in a particular society at the time of their use. After World War II during which gross violations of human rights were committed by the Nazis, the world entered the human rights era with the adoption of the Universal Declaration of Human Rights in 1948 [9]. The Declaration is consistent with the Western notion of liberal individualism. This culture of human rights gained huge ground in South Africa with the abolition of the apartheid regime and the drafting of the new Constitution of South Africa containing the Bill of Rights [10]. The Constitution aims to protect freedom of choice and individual rights such as the rights to Equality, Human Dignity, Life, Freedom and security of the person, Privacy, and Access to Health Care [11]. Respect for these rights is a vital part of ethical health care. The health disparities present in South Africa represent the legacy of colonialism and apartheid. The peaceful transition to democracy in South Africa represents the start of an interesting period within the larger context of Africa in its post-colonial era of independence. It is a time when a mixture of European and African ideologies guides policy and law in the country. On the one hand, there is the new Consumer Protection Act which lists the rights of the consumer. It reflects the Western notions of human rights and individualism. On the other hand, there is the third founding principle of the proposed National Health Insurance (NHI) Scheme in South Africa, which is the promotion of social solidarity through the equitable funding of health services [12]. This reflects the African communitarian principle of ‘ubuntu’ or people’s connectedness as fellow human beings. The Nguni saying, ‘Umuntu ngumuntu ngabantu’, meaning ‘a person is a person through other people’, aptly expresses this notion of the priority of community and interdependence [13]. Western biomedical, allopathic medicine represents the formal, professional health sector in the country, but African traditional, spiritualist medicine still plays an important role in the informal, folk sector. The South African Department of Health estimates that at least 70% of all South Africans consult traditional healers. For every 15 medical doctors, there are approximately 100 traditional health practitioners in the country [14].

Broadly speaking the above differences demonstrate the varying capitalist and socialist economic principles adopted in South Africa’s mixed economy. The particular political and economic systems present in a country play a major role in shaping its health care system, especially in terms of organisation and funding. The typology of William C Cockerham describes a fourfold classification of health care systems. It is a simplified classification that serves to illustrate certain principles inherent behind particular systems. The reality is much more complex. Most health care systems do not fall clearly into one category or the other, but may tend to be based on the underlying principles of a particular category.

The first type is free-market medicine. This is mainly based on free-market principles and minimal state intervention. It has a split system of health care delivery and financing – a private sector which is based on individual purchasing power, and a public sector based on welfare provision. Apart from being fragmented, these systems are usually highly inequitable. The second type is socialised medicine or the Beveridge model. This constitutes a state-supported service financed by taxation with little or no additional cost to the consumer. The government is directly involved and guarantees equal access to all citizens. The third is the Bismarck model which is manifested as decentralised national health programmes. Government control is more indirect and often functions as a third party between providers and financing organisations. The fourth is the Semashko model or socialist medicine as practised in communist or formerly communist countries. Health care is a state-provided public service with complete governmental control [3].

The introduction of the National Health Insurance (NHI) Scheme in South Africa will push the health care system towards a socialised model. NHI is a system of health care funding in which all tax-payers or income earners make mandatory contributions, but the whole population is entitled to the benefits, including those who do not contribute. The aim of the NHI is universal coverage of the population with adequate health care at an affordable cost. NHI promotes health risk cross-subsidisation across the whole population. The implementation of NHI will take place over the next 14 years. It began with the roll-out of pilot projects in 2012 [4,15-17].

### The changing view of the doctor-patient relationship

The consultation between doctor and patient is an integral part of medical practice and the formation of the doctor-patient relationship. Four models of this relationship have been described. Historically, the ‘paternalistic’ model was popular. This model is disease-focussed. The doctor is the expert (‘parent’) who makes decisions on behalf of the patient (‘child’). The ‘mutual’ relationship model is becoming more common now. It is based on the modern cultural belief that individuals should not be passive followers of authority, but rather self-determining free agents. The doctor recognises patient autonomy and adopts a ‘patient-centred’, bio-psychosocial approach. The ‘consumerist’ model has resulted in privatisation, patients ‘shopping around’, higher costs and a culture of litigation. However, it also promotes competition with resultant quality improvement and cost containment. The relationship is like a transaction between the consumer or purchaser (patient) and the seller, provider or supplier (doctor). Finally, the ‘default’ model is often found in situations where somatisation takes place. There is little engagement between the doctor and the patient [18].

In reality, modern medical practice calls for different models to be adopted in different contexts, as is appropriate to the particular clinical situation. There is fluidity present in the doctor-patient relationship, as we need to adapt to the complexity and diversity that characterise modern medicine. A paternalistic model may be more appropriate in the Emergency Department, whereas the mutual relationship model will often work best in a general practice context.

Various metaphors have been used to describe the doctor-patient relationship. Apart from ‘paternalism’ and ‘consumerism’ which are reflected in the four models described above, there are the metaphors of ‘education’ in which the doctor-patient relationship mimics teacher-scholar learning, ‘partnership’ which was alluded to with the label for a patient as ‘partner’, and ‘negotiation’ or the formation of a contract between two rational parties. A dominant metaphor in medical ethics and law today is the ‘fiduciary’ metaphor where the doctor is a regarded as a fiduciary for his patient. A fiduciary role relates to the Biblical concept of stewardship and originates from the law of trusts and agency. A fiduciary is defined as ‘someone with power or property to be used for the benefit of another and legally held to the highest standard of conduct’. It implies a relationship based on dependence and trust. A fiduciary may not promote his own interests or those of a third party. If he has divided loyalties or a conflict of interest, there is an increased risk of this ‘trust’ relationship being breached. There are many similarities between this relationship and the doctor-patient relationship. Ethical standards dictate that a doctor’s most important consideration should be the patient’s welfare. Legally, however, doctors are not held strictly accountable as fiduciaries. They may also have obligations to other parties such as medical aid schemes or managed care organisations (MCOs). Distributive justice and prioritisation of resources require an increasing emphasis on groups rather than individuals which implies an obligation to promote public health and consider all patients at any time, not just the specific, currently consulting patient [19].

The core fiduciary value of accountability remains essential in the doctor-patient relationship. In light of the recent National Health Amendment Bill No. 24 of 2011, accountability of individual health care providers and health workers, as well as collective ones (health establishments and facilities), will be enforced in South Africa through the establishment of the Office for Health Standards Compliance, as well as the appointment of an Inspectorate and an ombudsman by the Office’s Executive Director. The purpose of the Office is to make sure that complaints from health care users are investigated properly and dealt with promptly through an independent mechanism, as well as to facilitate compliance with the norms and standards of the national health system [20]. It echoes the spirit of the new Consumer Protection Act No. 68 of 2008 which, as the name suggests, aims to protect the consumer by enshrining fundamental consumer rights and establishing a National Consumer Commission, with a commissioner, inspectors and investigators [21].

### The ethical implications of the commodification of health care

If the patient is considered a ‘consumer’ of health care, there are various implications. The doctor takes on the role of ‘provider’ or ‘supplier’ of the ‘commodity’ or ‘product’ of health care. This role-shifting could result in the replacement of professional ethics with marketplace or business ethics. Plato makes it clear in The Republic that a doctor is firstly a healer of the sick, not a money-maker [22].

In a system of health financing where doctors are paid on a fee-for-service basis (the previous exclusive means of payment in the private sector of South African health care), the potential exists for doctors to over-service in order to generate additional income. Some corrupt doctors have even been caught claiming from medical insurance organisations more than once for a particular service rendered to a patient. In the system of managed care, which is playing an increasingly important role in the private sector of South African health care, a capitation system of payment is adopted. In this system, a specific amount is paid to the doctor per enrolled person for a set period of time. This incentivises under-provision of services by doctors because the more services they actually provide, the more a patient is costing them. Capitation is the method of payment that will be adopted in the new NHI in South Africa. Currently, public sector doctors are paid a set salary per month, regardless of number of patients seen or services provided. A system of managed care may encourage doctors to be motivated by financial rewards [23], however so can a fee-for-service model. Doctors need to guard against being motivated by money and becoming purely business-minded, at the expense of their patients.

The relationship between ethics and economics as well as the capitalist ethos is explored in Adam Smith’s ‘An Inquiry into the Nature and Causes of the Wealth of Nations’ (1776) and ‘The Theory of Moral Sentiments’ (1759) [24,25]. He argues that if everyone pursues his own interests, the interests of all will be served. The profit motive would ultimately lead to societal benefit. Smith did affirm that some things can only be assured by government intervention though. If health care were a mere commodity, the free market would be the best means of distribution of health care. However, health care is more than a commodity. A commodity is something a person possesses, and can sell or give away at his own free will. It implies exchangeability or interchangeability in terms of doctors, patients and the ‘product’ of care.

In certain contexts, this is acceptable within the health care system. A private sector patient will probably not mind going to any one of a number of interchangeable radiologists for a radiograph, as long as he obtains a product of adequate quality at a price that his medical insurance is willing to cover. In the public health sector of South Africa, there is such a high turnover of doctors that it is almost inevitable that doctors become interchangeable. In specific circumstances, a continuous doctor-patient relationship is definitely preferable, particularly in fields such as Family Medicine and Psychiatry.

A marketplace ethic would normally imply a more relaxed standard of truth-telling, where actions such as bluffing are allowed. Lying becomes more commonplace as each party (patient, doctor, and third party) tries to gain the maximum benefit for himself. Illingworth describes a ‘Warranty Theory of Truth’ which necessitates honesty in the context of the doctor-patient relationship where the patient’s health and life is at stake [26]. Section 41 of the Consumer Protection Act in South Africa deals with ‘False, misleading or deceptive representations’ [21]. Interestingly, it introduces a stringent form of business ethics that expressly prohibits defrauding or deception of customers in transactions. It attempts to introduce the ethic of veracity to all consumer transactions.

In Section 27(1)(a) of the South African Constitution, access to health care services, including reproductive health care, is listed as a right [11]. Section 27(2) makes it clear that the state must take reasonable measures within its available resources to ensure that this right is progressively realised. In other words, it is not something that can be taken away from someone. It is viewed as a universal human need. The poor and uninsured may not be deprived of health care due to lack of money to buy it. Health care is essential in the creation of health. Health is necessary for the fulfilment of human potential and to facilitate human thriving. Health care is not only for the individual good, but also for the common good [23]. It helps the family and community to bear the burden of disease and illness.

The professional ethic describes health care as a moral obligation in a good society where it represents a sense of caring for the community. It emphasises the principle of beneficence rather than just non-maleficence. Health care is patient-orientated and demands an altruistic, even self-effacing attitude. This is part and parcel of being a professional, in the traditional sense of the word. Traditionally, a profession is committed to providing services for the common good. Professionals have a ‘calling’ and are not motivated only by personal interest and financial gain. More recently, a professional is considered to be someone with autonomy who can exercise control over other occupations. There are many challenges to a doctor’s autonomy or freedom including the government and regulatory bodies [18]. The former definition is applicable in an ethical sense. Professionalism demands fidelity to the patient, regardless of self-interest [27]. Ill people are very vulnerable and therefore exploitable which means they need much more secure protection than the marketplace can provide, although the Consumer Protection Act attempts to create protection in this context. A degree of government intervention is still necessary to ensure equity and distributive justice. The South African government plans to create a more equitable health care system through the introduction of National Health Insurance [12,15-17].

### Increasing emphasis on the principle of respect for autonomy

In the traditional system of paternalism, beneficence - acting for the patient’s benefit - was the doctor’s main duty. Immanuel Kant criticised the ‘imperium paternale’ (the ‘paternalistic government’) in ‘The Critique of Pure Reason’ because he believed it restricted the freedom of its subjects [28]. The modern emphasis on liberal individualism and consumerism has placed patient and consumer rights at the fore. Autonomy, a person’s self-rule or self-governance, is now prioritised [29]. This goes hand-in-hand with the doctrine of ‘informed consent’. The principle of distributive justice becomes increasingly crucial when there is competition for scarce resources. Fair, equitable distribution of resources is particularly important in South Africa today considering that inequality and discrimination were rampant during apartheid. Vital to transformation and one of the aims of the new government is the redress of the imbalances of the past.

Autonomy applies to both patients and doctors. As mentioned previously, the doctor’s autonomy is limited by factors such as the control of authorities. Respect for patient autonomy and fundamental human rights is probably the value which has changed the most over the years. Patients, instead of doctors, are now the ultimate decision-makers with regards to their own bodies and health. Self-determination trumps the concept of the doctor, as an expert, knowing what’s best for the particular patient and making medical decisions on behalf of the patient [30]. Now the patient needs to make his own final decision about his care based on the options presented to him by the doctor. As mentioned previously, the doctor is no longer merely responsible for the individual patient. He has obligations to society including all other patients, as well as to third parties, regulatory authorities and the Law.

Sometimes, when taken to the extreme or abused, patient autonomy can become a problem. Patients who view themselves as informed ‘consumers’ in the medical market may demand medical services that the doctor views as inappropriate [30]. The twenty-first century being the era of information and technology also has a role to play. The Internet has resulted in certain patients, particularly in the private sector in South Africa, having easy access to information regarding health conditions. The reliability of such information is questionable as patients are not necessarily trained in the principles of critical appraisal and choosing peer-reviewed journals as sources for information. Nevertheless, patients today are definitely more likely to come armed with knowledge than patients in the past.

Another danger that has surfaced in countries where medical costs are exorbitant is that the savvy consumer may try to save money by turning to self-care in the form of internet-based, self-diagnosis and treatment in the form of medications acquired via online pharmacies. If not properly regulated, this is naturally a dangerous practice that could easily threaten a person’s well-being. Another possibility of abuse in the context of ‘medical consumerism’ exists in the field of plastic surgery. If patients are viewed as consumers, there might not be the same ethical obligation to refuse to do cosmetic surgery on patients with body dysmorphic disorder for whom no amount of refashioning will bring contentment with their image.

At the opposite end of the spectrum, there are patients who are unwilling to make decisions with regards to their own medical care. In developing or emergent countries like South Africa, there is still the problem of illiteracy with many uneducated or poorly educated patients being present. The challenge then becomes one of trying to empower the patient with knowledge and information through clear communication. Two major obstacles are the language and cultural barriers which often exist in a multicultural society [30]. The resources (e.g. health care educators, interpreters, doctor with training in an additional indigenous local language) are often not available to ensure that a patient is truly informed and able to exercise proper autonomy. The doctor is responsible for ascertaining if the patient truly understands the nature of the intended procedure, including risks and possible complications, and can give competent consent. Due to an extremely high patient burden in the public health sector, most doctors are not able to dedicate adequate time to ensure that poorly educated patients are properly informed in order to make their own decisions. Many of these patients seem to prefer the doctor to take on a paternalistic role and decide on their behalf, claiming ‘Doctor, you know best.’ Doctors, who are usually part of a wealthier, better educated socio-economic group, may view their low-income patients as incompetent to make certain health care decisions and find it easy to take on the rule of decision-maker. It is doubtful whether meaningful patient autonomy is possible in the setting of South Africa’s pervasive inequality.

Chapter 2 of The National Health Act No 61 of 2003 details the rights and duties of users (patients) and health care personnel. According to Section 6(1), users have the right to full knowledge of their health status, the range of options available to them, and the consequences of each option. They have the right to refuse health services. Section 6(2) states that the health care provider has the duty to inform the user in a language the user understands and in a way that takes the user’s level of literacy into account. Apart from certain special circumstances detailed in Section 7, a user must provide informed consent in order to receive a health service. Section 8(1) makes it clear that a user has the right to participate in any decision-making affecting his or her personal health and treatment [5]. Particularly in rural African settings, the communitarian ethic abounds and other members of the family or community may need to be consulted and give consent to the patient before he or she is able to make a health-related decision and give informed consent [13].

### The patient as a consumer in the current legal environment

The new South African Consumer Protection Act (CPA) and its regulations came into effect on 1 April 2011. According to Section 5(3), a regulatory authority may apply to the Minister of Trade and Industry for an industry-wide exemption from one or more provisions of this Act on the grounds that those provisions overlap or duplicate a regulatory scheme administered by that regulatory authority in terms of any other national legislation; or any treaty, international law, convention or protocol [21].

Thus far, only two regulatory bodies within the health care industry have formally applied for exemption. The Council for Medical Schemes (CMS) has applied for an industry-wide exemption from certain provisions of the CPA [31]. The South African Medical Device Industry Association (SAMED) has also applied for exemptions from certain provisions of the CPA on the basis of duplication of the existing regulatory framework. SAMED also claims that health care products are different to ‘other’ consumer goods because they carry an inherent risk of harm and do not always work, but these conditions are known and recorded by means such as package inserts. Most of the time, it is the health care professional who uses the device, not the consumer himself [32].

All patients are considered as ‘consumers’ from a legal perspective. This means patients also enjoy the rights of the consumer: the right to equality in marketing; the right to privacy; the right to choose; the right to disclosure of information; the right to fair and responsible marketing; the right to fair and honest dealing; the right to fair, just and reasonable terms and conditions; the right to fair value, good quality and safety; and the right to hold the supplier accountable [21]. Part F of the CPA describes the right to fair and honest dealings. Section 40 prohibits the use of coercion or other such means to convince a consumer or patient to accept a particular product or service. This will truly encourage the notion of patient autonomy and choice. Allowing the patient to make the decision and obtaining informed consent is vital because consumers have the right to choose. The consent should be truly ‘informed’ and this should be ensured by communication and documentation (including the informed consent form) in plain language easily understandable to the consumer. All agreements and policies such as a billing policy in private practice need to have fair terms and conditions as stipulated in the Act. The paying patient should also be informed of prices up front [33].

An important new development of the CPA is that it creates strict or no-fault liability for harm caused by goods. Section 61 explains that the producer or importer, distributor or retailer of any goods is liable for any harm caused by supplying unsafe goods; a product failure, defect or hazard in any goods; or inadequate instructions or warnings provided to the consumer about possible hazards. This is irrespective of whether harm resulted from any negligence [21]. In the medical context, goods could imply medications, implantations, and medical equipment. This has medico-legal implications for litigation, particularly in the private sector. Previously, medical malpractice suits were based on the common law principles of negligence. The injured patient had to prove that the doctor acted negligently in his provision of care, and that this negligence resulted in injury. This required that four legal elements be proven: 1) A professional duty owed to the patient (duty to care); 2) breach of such duty; 3) injury caused by the breach; and 4) resulting damages. These common law principles conform to the provisions of the National Health Act. However, the new CPA deviates from the common law and the National Health Act. Doctors can now not only be sued for negligence that resulted in harm to the patient, but also for supplying the patient with a faulty product that led to harm or loss [34].

With the patient being considered a ‘consumer’ in terms of the CPA, the doctor becomes a ‘supplier’ or ‘retailer’ as part of the health care supply chain. Section 61(3) of the CPA makes it clear that if, in a particular case, more than one person is liable, all the involved parties are jointly and severally liable [21]. This means that the patient can sue anyone in the supply chain. The doctor is placed in a difficult position because he or she is usually the only member of the supply chain who can be identified by the patient. The doctor is thus the easiest person to sue. Previously if a doctor was sued, common law dictated that the complainant had to prove negligence on the part of the doctor in order for his case to be successful. Now, however, due to the strict liability provisions in the CPA, this is no longer necessary. The only thing that will need to be established is the causal relationship between the harm suffered by the complainant and the product provided by the health-care practitioner on a balance of probabilities [35,36].

The importance of clear instructions and warnings to patients as well as drawing the patient’s attention to any unusual or serious risks and obtaining written consent cannot be over-emphasised if the doctor is to avoid litigation. Additionally, South African common law recognises the offence of injuria - the unlawful infliction of bodily harm or violation of physical integrity. Lack of consent therefore equates to assault [37]. Informed consent is not only a legal, but also an ethical imperative.

Section 54 of the CPA (‘Consumer’s rights to demand quality service’) affirms that the consumer has the right to the performance of services in a manner and quality that persons are generally entitled to expect, taking into account any specific conditions agreed upon before or during the performance. Due to the ‘Implied warranty of quality’ (Section 56), if the doctor does not fulfil these requirements, it may result in his having to fix the defect or refund the patient a reasonable share of the price paid. Section 47 of the CPA prohibits over-booking [21]. Doctors or hospitals cannot expect payment or any consideration for services they do not intend to supply as promised. This is particularly important in private hospitals with surgeons’ lists. Doctors who have made a commitment to provide a particular service at a certain time and on a specific date, and then fail to do so, run the risk of having to refund the patient [38].

Another avenue to assure quality of service is via the proposed Office of Health Standards Compliance. According to Donald Dinnie, a medico-legal attorney, the proposed amendments to the National Health Act will give patients at public health establishments a suitable means to complain and seek improvement of problem areas such as long waiting times, medication availability, safety and security, nursing attitude, values of staff, and infection prevention and control [personal communications]. The Consumer Protection Act complements the proposed amendments.

## Summary

It is interesting that on the one hand, South African legislature reflects the era of liberal individualism and human rights with its emphasis on the importance of informed consent and patient choice in the National Health Act and consumer rights in the Consumer Protection Act (CPA); yet, its plan to create National Health Insurance that promotes social solidarity is more a reflection of communitarian ethics and socialist principles. Ethically speaking, there are certain problems with considering patients as consumers of health care. Substituting professional ethics with marketplace ethics does not seem appropriate for an entity such as health care which is more than a mere commodity. There are detrimental implications for the doctor-patient relationship, a core component of health care. An advantage of considering patients as consumers is that it facilitates an emphasis on patient autonomy and the necessity of informed consent. The legal environment in South Africa at present with the new CPA and the proposed Office of Health Standards Compliance will both promote the protection of the patient’s rights as a consumer or user of health care. Although in a wealthier, first-world context, and perhaps the private sector in South Africa, the consumerist model could more easily lead to abuse and litigation, this is unlikely to occur in the developing context, such as the public health sector in South Africa. Here, the aim of the legislation is to protect the disadvantaged consumer from exploitation, and to empower resource-poor communities through increased levels of awareness and the creation of avenues to make their voices heard. Careful and ethical implementation will be necessary to ensure that this aim is achieved and that the consumer or patient does not become the exploiter.

## Abbreviations

CPA: Consumer Protection Act; MCOs: Managed Care Organisations; NHI: National Health Insurance; RSA: Republic of South Africa; SAMED: South African Medical Device Industry Association; USA: United States of America.

## Competing interests

The authors have no competing interests to declare.

## Authors’ contributions

KR wrote the article, revised it during the pre-publication process and compiled the literature. KM was involved in the conception of the topic and edited and mentored the revision of the article for publication. Both authors read and approved the final manuscript.

## Authors’ information

KR originally wrote this article as an entry for the 2011 Ethics Essay Writing Competition sponsored by the Medical Protection Society (MPS) and organised by the Centre for Medical Ethics and Law for medical students at Stellenbosch University. The topic provided was ‘Patients as consumers of health care: the ethical and legal implications’. Hers was the winning entry and she received R5000 prize money at the MPS Annual Ethics Conference ‘Ethics for All’ hosted at the Cape Town International Convention Centre (CTICC) in November 2011.

KM [MB ChB, MFamMed, MPhil (Applied Ethics), FCFP (SA), DPhil] is the Associate Professor and Head of the Centre for Medical Ethics and Law (CMEL) at Stellenbosch University. She is recognised as a leading authority in the field of Bioethics and serves on South Africa's National Health Research Ethics Council (NHREC) in Pretoria. She is also a member of the following: the HIV Preventive Research Data Safety Monitoring Board (DSMB) in the Division of AIDS, National Institutes of Health (NIH), Washington DC, USA; the Scientific Review Committee, European and Developing Countries Clinical Trial Partnership (EDCTP), The Hague, Netherlands; the Board of Directors, South African Medical Research Council (MRC), Cape Town; SAGE Working Group on Immunisation in Humanitarian Crises, World Health Organisation (WHO), Geneva, Switzerland.

## Pre-publication history

The pre-publication history for this paper can be accessed here:

http://www.biomedcentral.com/1472-6939/14/15/prepub
